# Biomarkers for immune-related adverse events in cancer patients treated with immune checkpoint inhibitors

**DOI:** 10.1093/jjco/hyad184

**Published:** 2024-01-05

**Authors:** Yao Liang, Osamu Maeda, Yuichi Ando

**Affiliations:** Department of Clinical Oncology and Chemotherapy, Nagoya University Hospital, Nagoya, Aichi, Japan; Department of Clinical Oncology and Chemotherapy, Nagoya University Hospital, Nagoya, Aichi, Japan; Department of Clinical Oncology and Chemotherapy, Nagoya University Hospital, Nagoya, Aichi, Japan

**Keywords:** immune checkpoint inhibitor, immune-related adverse event, biomarker

## Abstract

Although immune checkpoint inhibitors have greatly improved cancer therapy, they also cause immune-related adverse events, including a wide range of inflammatory side effects resulting from excessive immune activation. Types of immune-related adverse events are diverse and can occur in almost any organ, with different frequencies and severities. Furthermore, immune-related adverse events may occur within the first few weeks after treatment or even several months after treatment discontinuation. Predictive biomarkers include blood cell counts and cell surface markers, serum proteins, autoantibodies, cytokines/chemokines, germline genetic variations and gene expression profiles, human leukocyte antigen genotype, microRNAs and the gut microbiome. Given the inconsistencies in research results and limited practical utility, there is to date no established biomarker that can be used in routine clinical practice, and additional investigations are essential to demonstrate efficacy and subsequently facilitate integration into routine clinical use.

## Teaser

There is to date no established biomarker for immune-related adverse events resulted from immune checkpoint inhibitors in routine clinical practice, and further investigations are indispensable to validate efficacy and accuracy.

## Introduction

In recent years, there has been considerable progress in cancer treatment through use of immune checkpoint inhibitors (ICIs). The Pharmaceuticals and Medical Devices Agency in Japan has approved several such agents for various cancers. These include anti-cytotoxic T-lymphocyte antigen 4 (anti-CTLA-4) antibodies (ipilimumab and tremelimumab), anti-programmed death 1 (anti-PD-1) antibodies (nivolumab, pembrolizumab and cemiplimab) and anti-programmed death 1 ligand (anti-PD-L1) antibodies (durvalumab, atezolizumab and avelumab). Cytotoxic T cells (CTLs) express inhibitory receptors, including CTLA-4 and PD-1, that interact with cluster differential 80 (CD80)/cluster differential 86 (CD86) and PD-L1 expressed on antigen-presenting or tumor cells. These interactions lead to suppression of the T-cell immune response and help tumor cells to evade T-cell-mediated cell death (1,2). ICIs targeting the CTLA-4 or PD-1/PD-L1 axis prevent binding between these ligands and receptors from impeding signal transduction and therefore enhance the antitumor immune response.

Although use of ICIs has lead to favorable clinical outcomes, it also results in a broad range of inflammatory side effects due to excessive immune activation, known as immune-related adverse events (irAEs) (3). Although the precise mechanisms of irAEs are still unknown, several potential mechanisms include heightened T-cell activity targeting antigens presented in both tumor and healthy tissues, along with escalated inflammatory cytokine levels. Types of irAEs are diverse and can occur in almost any organ and tissue, with different frequencies and severities (4). The most common irAEs often involve the skin, gastrointestinal tract, endocrine glands and liver; they are often difficult to treat and might worsen quality of life (QoL) (5). Pneumonitis and myocarditis are rare, occurring in 0.3 to 1.3% of cases, but can be fatal (6). Furthermore, irAEs may occur within the first few weeks after treatment or even several months after treatment discontinuation (3,7). Given the variation of organs and tissues of origin, severity, and uncertainty of onset, biomarkers for predicting irAEs and managing them as early as possible are highly desired, helping to maintain effective treatment of patients and their QoL.

**Figure 1:**
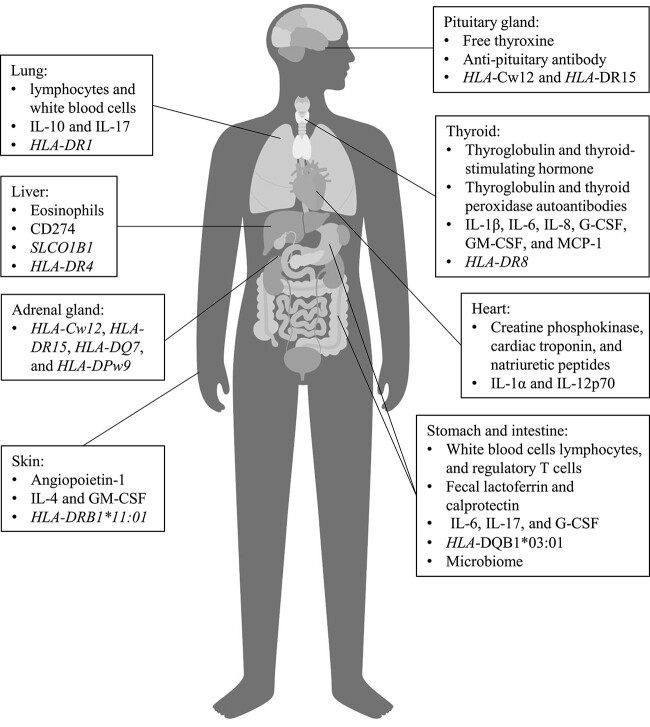
Biomarkers for organ-specific irAEs. Abbreviations: irAEs, immune-related adverse events; HLA, human leukocyte antigen; IL, interleukin; G-CSF, granulocyte colony-stimulating; GM-CSF, granulocyte-macrophage colony-stimulating; MCP-1, monocyte chemoattractant protein-1.

There are currently no definitive biomarkers for routine clinical practice, and efficacy and accuracy will need to be validated. Here, we review the literature on various types of potential predictive biomarkers for irAEs (Figure 1).

### Blood cell counts and cell surface markers

Because routine laboratory tests of blood counts and blood cell ratios are easily accessible, inexpensive and simple, clinicians are inclined to use them as biomarkers for irAEs. However, most studies to date are retrospective, had inconsistent cutoff values, and may contain many biases, and the results were difficult to interpret (Table 1).

**Table 1:** Blood cell count and ratio biomarkers for predicting irAEs

Parameter	Study design (no. patients)	Tumor type	irAE type	Association
Cell counts				
WBC count	Retrospective (*n* = 101)	Melanoma	Lung and gastrointestinal irAEs	High WBC counts after treatment were associated with high risk of grade 3–4 lung or gastrointestinal irAEs (OR, 1.20; *P* = 0.014) (8)
Neutrophil count	Retrospective (*n* = 150)	NSCLC	All	Low absolute neutrophil counts at baseline and when irAEs occurred were associated with high risk of grade 3–4 irAEs (univariate analysis: *P* = 0.009; *P* = 0.000281, respectively) (9)
Neutrophil-to-lymphocyte ratio	Retrospective (*n* = 150)	NSCLC	All	Low neutrophil-to-lymphocyte ratio at baseline and when irAEs occurred were associated with high risk of grade 3–4 irAEs (univariate analysis: *P* = 0.023; *P* = 0.011, respectively) (9)
	Retrospective (*n* = 470)	Various cancers	All	Baseline neutrophil-to-lymphocyte ratio ≤ 5.3 was associated with high risk of any-grade irAEs (OR, 2.07; *P* = 0.01) (10)
	Prospective (*n* = 1187)	Solid and blood cancers	All	High baseline neutrophil-to-lymphocyte ratio was associated with high risk of grade 4 or worse irAEs (univariate analysis: *P* = 0.0137) (11)
Eosinophil count	Retrospective (*n* = 105)	Various cancers	All	High baseline absolute eosinophil counts >0.175 × 10^9^/L were associated with high risk of any-grade irAEs (OR, 0.29; *P* = 0.02) (12)
	Retrospective (*n* = 533)	Solid tumors	Liver injury	Baseline absolute eosinophil counts ≥130 μ/L were associated with high risk of grade 2 or worse immune-related liver injury (HR, 3.01; *P* = 0.012) (13)
Eosinophil	Retrospective (*n* = 149)	NSCLC	All	Baseline percentage of eosinophil ≥1.15 was associated with high risk of any-grade irAEs (OR, 8.30; *P* = 0.003) (14)
Monocyte count	Retrospective (*n* = 470)	Various cancers	All	Baseline absolute monocyte counts >0.29 × 10^3^/μL were associated with high risk of any-grade irAEs (OR, 2.34; *P* = 0.03) (10)
Monocyte-to-lymphocyte ratio	Retrospective (*n* = 470)	Various cancers	All	Baseline monocyte-to-lymphocyte ratio ≤ 0.73 was associated with high risk of any-grade irAEs (OR, 2.96; *P* = 0.001) (10)
Lymphocyte count	Retrospective (*n* = 470)	Various cancers	All	Baseline absolute lymphocyte counts >2.6 × 10^3^/μL were associated with high risk of any-grade irAEs (OR, 4.30; *P* = 0.002) (10)
	Retrospective (*n* = 171)	NSCLC	All	Absolute lymphocyte counts >820/mm^3^ at 2 weeks after treatment initiation were associated with high risk of any-grade irAEs (OR, 3.58; *P* = 0.007) (15)
	Retrospective (*n* = 226)	Solid tumors	All	Low absolute lymphocyte counts when irAEs occurred were associated with high risk of grade 3–4 irAEs (univariate analysis: *P* = 0.005) (16)
	Retrospective (*n* = 101)	Melanoma	Lung and gastrointestinal irAEs	High relative lymphocyte counts after treatment were associated with high risk of grade 3–4 lung or gastrointestinal irAEs (OR, 1.65; *P* = 0.012) (8)
	Retrospective (*n* = 105)	Various cancers	All	Low baseline relative lymphocyte counts were associated with high risk of any-grade irAEs (OR, 3.6; *P* = 0.027) (12)
Platelet count	Retrospective (*n* = 470)	Various cancers	All	Baseline platelet counts >145 × 10^9^/L were associated with high risk of any-grade irAEs (OR, 2.23; *P* = 0.03) (10)
	Retrospective (*n* = 150)	NSCLC	All	Low platelet counts at baseline were associated with high risk of grade 3–4 irAEs (univariate analysis: *P* = 0.023) (9)
	Retrospective (*n* = 105)	Various cancers	All	Low baseline platelet counts were associated with high risk of any-grade irAEs (OR, 2.77; *P* = 0.025) (12)
Platelet-to-lymphocyte ratio	Retrospective (*n* = 150)	NSCLC	All	Low platelet-to-lymphocyte ratio at baseline and when irAEs occurred were associated with high risk of grade 3–4 irAEs (univariate analysis: *P* = 0.0016) and grade 1–2 irAEs (univariate analysis: *P* = 0.011), respectively (9)
	Retrospective (*n* = 470)	Various cancers	All	Baseline platelet-to-lymphocyte ratio ≤ 534 was associated with high risk of any-grade irAEs (OR, 5.05; *P* = 0.04) (10)
	Retrospective (*n* = 184)	NSCLC	All	Baseline platelet-to-lymphocyte ratio < 180 was associated with high risk of any-grade irAEs (OR, 2.3; *P* = 0.027) (17)
Cell surface markers				
CD8^+^ T-cell	Retrospective (*n* = 40)	Various cancers	All	Low baseline CD8^+^ T-cell counts were associated with high risk of any-grade irAEs (OR, 0.934, *P* = 0.012) (12)
Regulatory T-cell	Prospective (*n* = 26)	Melanoma	Colitis	Low baseline median regulatory T-cell proportion was associated with high risk of any-grade colitis (univariate analysis: *P* = 0.018) (19)
CD4 memory T-cell and T-cell receptor	Retrospective (*n* = 71)	Melanoma	All	Baseline activated CD4 memory T-cell abundance and T-cell receptor diversity correlated with severe irAE development; increased T-cell receptor clonality during treatment correlated with severe irAE development and early irAE onset time (20)
CD19^+^ B-cell	Retrospective (*n* = 40)	Various cancers	All	High baseline proportion of CD19^+^ B cells was associated with high risk of any-grade irAEs (OR, 15.87, *P* = 0.02) (12)
CD21^lo^ B-cell	Prospective (*n* = 23)	Renal cell carcinoma	All	Low baseline proportion of CD21^lo^ B cells was associated with high risk of any-grade irAEs (univariate analysis: *P* < 0.01) (21)

Note: Data were calculated by multivariate analysis, unless otherwise specified; *P* < 0.05 was statistically significant.

Abbreviations: irAE, immune-related adverse event; WBC, white blood cell; OR, odds ratio; NSCLC, non-small cell lung cancer; HR; hazard ratio.

Many studies have shown that blood cell counts and ratios might be predictive factors of irAE occurrence (8–17). Most indicate that elevated blood cell counts, especially lymphocyte counts and a corresponding decrease in the individual blood cell-to-lymphocyte ratio, at baseline are associated with increased risk of irAEs. However, as is often the case, there are conflicting and inconsistent reports (9,11,12,16). Of particular interest, in a retrospective study of patients with solid tumors, those who experienced grade 3 or higher irAEs had significantly decreased lymphocyte counts at irAE onset (8–12,15,16), suggesting that changes in blood counts during treatment, as well as at baseline, may be important.

It has been reported that ipilimumab leads to increased T-cell repertoire diversification before irAE onset, with greater diversity of CD4^+^ and CD8^+^ T-cells in patients with irAEs compared with those without irAEs (18), suggesting that subtypes of T-cells might act as biomarkers to predict the occurrence of irAEs. A retrospective analysis suggested that a low level of CD8^+^ T-cells at baseline predicts the occurrence of irAEs (odds ratio (OR), 0.934, 95% confidence interval (CI) 0.885–0.985, *P* = 0.012) (12). Similarly, patients with melanoma experiencing ipilimumab-related colitis tend to have a high level of CD4^+^ T-cells (*P* = 0.053) and a low proportion of regulatory T-cells (*P* = 0.018) at baseline (19). In contrast, no significant difference in absolute regulatory T-cell counts at baseline between groups with or without colitis has been reported. A study on T-cell characteristics associated with irAEs in patients with melanoma reported that activated CD4 memory T-cell abundance and T-cell receptor diversity at baseline correlated with severe irAE development. In addition, increased T-cell receptor clonality during treatment was associated with severe irAE development and onset time (20). In addition, baseline levels of several subtypes of B cells, such as CD19^+^ B-cells and B-cells with low CD21 expression (CD21^lo^ B-cells), are significantly associated with high risk of irAEs (12,21).

### Serum proteins

Similar to blood counts, serum proteins are measured by routine laboratory tests and may be promising biomarkers for both general and organ-specific irAEs. However, these protein levels are susceptible to many factors, including cancer-related inflammation and cancer development, which inevitably limits their specificity as biomarkers.

C-reactive protein (CRP) has been well studied, but results are inconsistent. A retrospective cohort study of 67 patients with primary liver cancer found a transient increase in CRP upon irAE onset; at baseline, however, there was no difference between patients with any grade irAEs and those without (22). Another retrospective study of 37 patients with melanoma treated with ICIs and tyrosine kinase inhibitors showed that CRP dramatically increased in the majority of patients (93%) when irAEs occurred (23). Nevertheless, it has also been reported that a low level of pretreatment CRP is independently associated with irAE occurrence (24). Similarly, results for other serum proteins, such as lactate dehydrogenase and albumin, are also inconsistent (12,25,26).

Some proteins may function as biomarkers of organ-specific irAEs. For example, higher levels of creatine phosphokinase, aspartate aminotransferase (AST) and alanine aminotransferase (ALT) after ICI therapy initiation correlate well with greater risk of ICI-induced myocarditis (27), and cardiac troponin and natriuretic peptides are frequently elevated when ICI-induced myocarditis is diagnosed (28). In a review of ICI-mediated diarrhea and colitis, fecal lactoferrin and calprotectin were reported to serve as useful surrogate markers in patients with ICI-mediated diarrhea and colitis, as in those with inflammatory bowel disease (29). In addition, decreased free thyroxine after ICI therapy initiation has been suggested to be an early biomarker of ICI-induced hypophysitis (30). Elevated levels of thyroglobulin after ICI therapy initiation and thyroid-stimulating hormone at baseline have been associated with ICI-induced thyroid dysfunction (31–33).

### Autoantibodies

Some organ-specific autoantibodies are often associated with irAEs in relevant organs; thus, the presence of autoantibodies at baseline is associated with the incidence or severity of irAEs associated with that organ. However, rather than predicting the occurrence of irAEs, autoantibodies may simply contribute to preemptive diagnosis of an immune-related disease present prior to ICI therapy. Further investigation is needed to clarify the association between autoantibody detection and irAE development.

Many studies have demonstrated the association between preexisting autoantibodies and the occurrence of irAEs in patients with multiple cancers (32,34–38). For instance, patients positive for anti-nuclear (ANA) or anti-thyroid antibodies at baseline might be at higher risk of developing organ-nonspecific irAEs than those without (34,38). Anti-pituitary antibodies and anti-thyroid antibodies are positively associated with adrenocorticotropic hormone (ACTH) deficiency and thyroid dysfunction, respectively (39–41). Pituitary endocrine cells express CTLA-4, which can be blocked by CTLA-4 inhibitors, leading to activation of complement components and pituitary gland damage (42). ICI-induced thyroiditis is a T lymphocyte-mediated process with elevated intrathyroidal CD8^+^ and CD4^−^CD8^−^ T lymphocytes (43). A possible interpretation is that T cells strengthen PD-1 antibody efficacy and, in turn, induce B cells to produce autoantibodies, leading to irAE occurrence and indicating that the presence of preexisting autoantibodies predicts irAEs (44). In addition to levels at baseline, obvious titer increases in thyroglobulin and thyroid peroxidase autoantibodies after starting ICI therapy and rheumatoid factor positivity at any time correlate with thyroid dysfunction and rheumatic irAEs (31,45).

Conversely, some studies have found that preexisting antibodies do not increase risk of irAE occurrence (46,47). Indeed, one retrospective study demonstrated no association between those baseline antibody titers and irAE severity (46). It has also been reported that although preexisting ANA at baseline is not associated with irAE rates, its appearance correlates positively with irAE severity (47).

In a retrospective analysis involving pretreatment serum samples from 333 patients with metastatic melanoma undergoing ICI therapy, it was observed that heightened levels of anti-MAGEB4 antibodies correlate with increased incidences of irAEs but that elevated anti-FGFR1 antibody levels are linked to decreased irAE occurrences (48).

There are both advantages and disadvantages when considering the use of ICI therapy in patients known to be autoantibody positive or diagnosed with an autoimmune disease. Historically, such patients were routinely excluded from clinical trials, and safety data were limited (49). However, recent reports have indicated that the safety of ICI treatment in these patients is comparable with that of others (50,51). In addition, studies have suggested that a combination of immunosuppressants can be safely used alongside ICI treatment without compromising its effectiveness (50). Therefore, we believe that ICI therapy can be considered a viable option for patients known to be autoantibody positive or diagnosed with an autoimmune disease.

### Cytokines/chemokines

Cytokines and chemokines play a crucial role in multiple inflammatory reactions and the tumor microenvironment (52,53). Therefore, they might serve as promising biomarkers for irAEs. However, findings regarding the correlation between cytokines/chemokines and occurrence of irAEs are currently vary and are inconsistent. Many studies have investigated and demonstrated the value of specific cytokines that can amplify both pro- and anti-inflammatory immune responses as predictive biomarkers for identifying an individual’s risk of developing irAEs (53). Among pro-inflammatory cytokines, the interleukin-1 (IL-1) family is associated with innate immune responses, with IL-1α and IL-1β exhibiting the highest inflammatory activity (54). A retrospective study showed that an elevated baseline level of IL-1α was significantly associated with the incidence of myositis in advanced gastrointestinal cancer patients (55). In another prospective study focusing on biomarkers of irAEs in non-small cell squamous carcinoma (NSCLC) patients, the occurrence of irAEs correlated with a higher baseline level of IL-1β (56). Similarly, concerning organ-specific irAEs, it has been suggested that the baseline level of IL-1β is significantly higher in those with than without thyroid dysfunction (31).

As a proinflammatory cytokine, systemic release of IL-6 has been implicated in the mechanism driving irAEs (57). In line with this, it has been proposed that a higher baseline level of IL-6 can be considered a risk factor for organ-specific irAEs, including thyroiditis and colitis (55). However, a different study involving melanoma patients reported the opposite finding, namely, that a lower baseline level of IL-6 correlated significantly with higher risk of irAEs (58). When combined with CRP, an increased level of IL-6 is considered a screening parameter for early detection of irAEs (59).

Through analysis of cytokine expression in melanoma patients experiencing irAEs, it was discovered that patients who develop pneumonitis and grade 3 gastrointestinal irAEs have an increased baseline level of IL-17 (60,61). There are several other proinflammatory cytokines that may serve as biomarkers for irAEs. For instance, higher baseline levels of IL-12p70 and leukemia inhibitory factor (LIF) are associated with myositis, granulocyte-macrophage colony-stimulating factor is linked to rash, and interferon-gamma (IFN-γ) is relevant to various irAEs (14,55,56). Conversely, it was revealed that a low baseline level of granulocyte colony-stimulating factor and an early decrease in IL-8 are significantly linked to the occurrence of irAEs such as thyroid dysfunction and colitis (31,60).

As typical anti-inflammatory cytokines, IL-4 and IL-10 have value as risk factors for irAE occurrence. It was shown that patients with ICI-related rash had higher baseline levels of IL-4 than those without this irAE (55). Moreover, elevated levels of IL-10 both at baseline and after the first cycle of immunotherapy correlated positively with onset of irAEs, especially in the case of pneumonitis (56,62).

As a category of cytokines capable of causing directional cell movement, chemokines are potential biomarkers for predicting irAE occurrence. For example, compared with patients who did not experience any neurological irAEs, those with high-grade irAEs showed an increased baseline level of monocyte chemoattractant protein-1 (MCP-1) (63). Furthermore, a significant association was observed between an early decrease in MCP-1 level during treatment and occurrence of thyroid irAEs (31). A longitudinal analysis of the relationship between cytokines/chemokines and irAEs revealed low baseline levels of C-X-C motif chemokine ligand 9 (CXCL9), CXCL10, CXCL11 and CXCL19 in patients who experienced irAEs. In addition, these patients had large increases in CXCL9 and CXCL10 levels after treatment (64). CXCL9, CXCL10 and CXCL11 are chemotactic factors for activated T cells and involved in T-cell recruitment, which indicates that elevated levels of these chemokines might lead to broad T-cell activation, thereby inducing irAE occurrence. CXCL13, functioning as a B-cell chemoattractant and playing a role in multiple autoimmune and inflammatory diseases, shows a significant association with irAE occurrence, whereby high baseline and treatment-related levels of CXCL13 are significantly linked to irAE occurrence (65).

Furthermore, there are additional potential cytokine/chemokine-related biomarkers capable of predicting the risk of irAEs. These include high baseline levels of angiogenin, angiopoietin-1, CD40L and B-cell-activating factor as well as increased levels of regulated upon activation, normal T cell expressed and secreted (RANTES) and soluble cluster of differentiation 163 during treatment (56,60,66–68).

### Genetic variations and gene expression profile

Because gene variants and differential gene expression can contribute to susceptibility to irAEs, there is great promise in utilizing multigene panels to assess an individual’s risk of developing irAEs.

Several studies have highlighted the association between single-nucleotide polymorphisms (SNPs) in the PD-1 protein-encoding gene **programmed cell death 1* (*PDCD1*)* and the frequency and severity of irAEs (Table 2). For example, among patients with NSCLC, those who carry the homozygous *PDCD1* 804C > T (rs2227981) gene variant were found to be less likely to experience irAEs of any grade (69). Conversely, patients with metastatic renal cell carcinoma carrying the *PD-1.6* G allele (rs10204525) are more prone to developing more severe and diverse irAEs than those with the AA genotype (70). In a genome-wide study, 12 SNPs were identified as risk factors for irAE occurrence; 18 other SNPs were protective factors (71). It has been suggested that various genetic alterations, including small variations and copy number variations in genes, such as *SMAD family member 3* (*SMAD3*), *PR/SET domain 1* (*PRDM1*), *interleukin 1 receptor antagonist* (*IL1RN*), *CD274*, *solute carrier organic anion transporter family member 1B1* (*SLCO1B1*), *thyroid stimulating hormone receptor* (*TSHR*) and *FANCD2 and FANCI associated nuclease 1* (*FAN1*), are associated with irAEs, especially some organ-specific irAEs, including hepatitis and encephalitis (72).

**Table 2:** Gene-related biomarkers for predicting irAEs

Gene	Study design (no. patients)	Tumor type	irAE type	Association
Genetic variation				
*PDCD1*	Prospective (*n* = 322)	NSCLC	All	The homozygous genotype *PDCD1* 804C > T (rs2227981) was associated with low risk of any-grade irAEs (OR, 0.4; *P* = 0.039) (69)
*PDCD1*	Retrospective (*n* = 106)	Renal cell carcinoma	All	The *PD-1.6* G allele of *PDCD1* (rs10204525) was associated with high risk of grade 2 or worse (OR, 3.39; *P* = 0.003) and more various (OR, 2.778; *P* = 0.031) irAEs (70)
*Spleen associated tyrosine kinase* (*SYK*)	Prospective (*n* = 95)	Melanoma	All	The *SYK* T/T genotype (rs7036417) was associated with high risk of grade 3 or 4 irAEs (OR, 7.46; *P* = 0.000143) (94)
*IL-17*	Prospective (*n* = 214)	Melanoma	All	The A allele of *IL-17* (rs16906115) and was associated with high risk of grade 3 or worse irAEs (OR, 2.24; *P* = 0.046) (95)
*Uracil DNA glycosylase* (*UNG*), *interferon omega 1* (*IFNW1*), *PD-L1*, *interferon lambda 4* (*IFNL4*) and *CTLA4*	Retrospective (*n* = 94)	Various cancers	All	*UNG* (rs246079), *IFNW1* (rs10964859), *PD-L1* (rs4143815), *IFNL4* (rs12979860) and *CTLA4* (rs3087243) were significantly associated with irAEs (univariate analysis) (96)
*Mitogen-activated protein kinase 1 *(*MAPK1*), *protein tyrosine phosphatase receptor type C* (*PTPRC*), *adenosine deaminase domain containing 1* (*ADAD1*) and *IL6*	Prospective (*n* = 340)	Solid tumors	All	The C allele of *MAPK1* (rs3810610) was associated with high risk of any-grade irAE (OR, 1.495; *P* = 0.012); the A allele of *PTPRC* (rs6428474) was associated with low risk of any-grade irAE (OR, 0.717; *P* = 0.041); the A allele of *ADAD1* (rs17388568) was associated with high risk of severe irAEs (OR, 2.599; *P* = 0.003); the G allele of *IL6* (rs1800796) was associated with low risk of severe irAEs (OR, 0.425; *P* = 0.018) (97)
Multiple genes	Retrospective (*n* = 89)	Melanoma	All	12 SNPs were risk factors for irAE occurrence; 18 SNPs were protective (71)
*SMAD3, PRDM1*, *IL1RN*, *CD274*, *SLCO1B1, TSHR* and *FAN1*	Prospective (*n* = 95)	Melanoma	All	VARs of *SMAD3* were associated with pancreatitis; CNVs of *PRDM1* and *IL1RN* were associated with irAEs; CNVs of *CD274* and *SLCO1B1* were associated with hepatitis; CNVs of *PRDM1* and *CD274* were associated with encephalitis; CNVs of *PRDM1*, *CD274*, *TSHR* and *FAN1* were associated with myositis (univariate analysis) (72)
Gene expression				
*LCP1* and *ADPGK*	Retrospective (*n* = 18 706)	Various cancers	All	High expression of *LCP1* (*P* = 0.008) and *ADPGK* (*P* = 0.01) were associated with any-grade irAEs (tumor tissues; univariate analysis) (73)
*CD3 epsilon subunit of T-cell receptor complex* (*CD3E*), *interleukin 2 receptor subunit gamma* (*IL2RG*), *CD4*, *CD37* and *IL-32*	Prospective (*n* = 162)	Melanoma	Gastrointestinal irAEs	High baseline expression of *CD3E*, *IL2RG*, *CD4*, *CD37* and *IL-32* was associated with any-grade irAEs (blood samples; repeated measures analysis of variance) (74)
**C-X-C motif chemokine receptor 1* (*CXCR1*)*	Retrospective (*n* = 355)	Melanoma	All	High expression of *CXCR1* pre- (*P* = 0.0034) and posttreatment (*P* < 0.001) was associated with low risk of irAEs (blood samples; univariate analysis) (75)
16 genes	Prospective (*n* = 150)	Melanoma	Diarrhea/Colitis	Variations in expression levels of 16 specific genes were associated with diarrhea/colitis severity (blood samples; univariate analysis) (76)
*HLA*				
*HLA*-Cw12, *HLA*-DR15, *HLA*-DQ7 and *HLA*-DPw9	Retrospective (*n* = 62)	Various cancers	Endocrine irAEs	*HLA*-Cw12, *HLA*-DR15, *HLA*-DQ7 and *HLA*-DPw9 were associated with isolated adrenocorticotropic hormone deficiency; *HLA*-Cw12 and *HLA*-DR15 were associated with hypophysitis (univariate analysis: all *P* < 0.05) (39)
*HLA-*DRB1*11:01 and *HLA-*DQB1*03:01	Prospective (*n* = 102)	NSCLC and melanoma	All	*HLA*-DRB1*11:01 was associated with pruritus (OR, 4.53; *P* = 0.0021), and *HLA*-DQB1*03:01 was associated with colitis (OR, 3.94; *P* = 0.017) (78)
*HLA*-B*27:05	Prospective (*n* = 5)	Breast and bladder cancers	Encephalitis	*HLA*-B*27:05 was associated with encephalitis (OR, 59.1; *P* < 0.001) (79)
*HLA*-DR1, *HLA*-DR4, *HLA*-DR8 and *HLA*-DR15	Retrospective (*n* = 132)	Melanoma	All	*HLA*-DR1 was associated with pneumonitis, *HLA*-DR4 was associated with type 1 diabetes and hepatitis, *HLA*-DR8 was associated with hypothyroidism, and *HLA*-DR15 was associated with hypophysitis (univariate analysis: all *P* < 0.01 except for hepatitis: *P* < 0.05) (80)
*HLA*-DRB1*04:01, *HLA*-DQB1*03:01 and *HLA*-DRB1*15:01	Prospective (*n* = 179)	NSCLC	All	*HLA*-DRB1*04:01 was associated with high risk of irAEs (RR, 1.55; *P* = 0.011; *HLA*-DQB1*03:01 was associated with low risk of colitis (RR, 0.18; *P* = 0.029), and *HLA*-DRB1*15:01 was associated with low risk of arthralgia (*P* = 0.048) (81)
*HLA*-DRB3*01:01, *HLA*-DPB1*04:02 and *HLA*-A*26:01	Retrospective (*n* = 530)	Various cancers	All	*HLA*-DRB3*01:01 was associated with thrombocytopenia (OR, 3.48; *P* = 0.011); *HLA*-DPB1*04:02 was associated with hypokalemia/hyponatremia (OR, 3.44; *P* = 0.009), leukopenia (OR, 2.1; *P* = 0.037), and anemia (OR, 2.33; *P* = 0.026); *HLA*-A*26:01 was associated with bilirubin elevation (OR, 2.67; *P* = 0.037) (82)

Note: Data were calculated by multivariate analysis, unless otherwise specified.

Abbreviations:VARs, small variations; CNVs, copy number variations; HLA, human leucocyte antigen; RR, relative risk.

Specific gene expression signatures also serve as valuable biomarkers for irAEs (73–76). In a notable example, a large-scale retrospective multiomics analysis involving various cancers revealed that patients who experience irAEs show high expression levels of certain genes. One such gene is **lymphocyte cytosolic protein 1* (*LCP1*)*, which encodes lymphocyte cytosolic protein 1 and is involved in T-cell activation. Another gene is **adenosine diphosphate dependent glucokinase* (*ADPGK*)*, encoding adenosine diphosphate–dependent glucokinase and playing a role in mediating metabolic shifts during T-cell activation (73). These gene expression patterns provide valuable insights into the underlying mechanisms of irAEs and may serve as predictive markers for their occurrence.

### Human leucocyte antigen genotypes

Human leukocyte antigen (HLA) molecules are expressed on the surface of immune cells and play a crucial role in presenting peptide ligands to T-cell receptors. With a growing body of research indicating associations between certain *HLA* genotypes and organ-specific irAEs, it is reasonable to view *HLA* genotype as a potential biomarker for predicting organ-specific irAEs.

*HLA* genes are known to be highly polymorphic, and certain specific variations in these genes can act as biomarkers for autoimmune diseases (77). Similar to autoimmune diseases, it has been suggested that *HLA* gene variations may influence the occurrence of irAEs. This viewpoint is supported by multiple studies that have identified significant associations between specific *HLA* variations and irAEs, especially in the context of some organ-specific irAEs (Table 2) (39,78–82). For example, it has been reported that patients with HLA-DR15 are at a high risk of experiencing isolated ACTH deficiency and hypophysitis (39,80). Indeed, the relationship between HLA-DQB1*3:01 and colitis appears to be contradictory. One study suggested that patients with HLA-DQB1*3:01 are more susceptible to developing colitis (78), whereas another study indicated that the same genotype might offer protection against colitis (81). This highlights the complexity of the relationship between HLA gene variations and irAEs, underlining the need for further research to refine these associations for clinical application.

### microRNAs

Although microRNAs (miRNAs) have gained interest in recent years, only a limited number of studies have established a significant association between specific miRNAs and irAEs. Indeed, this area of research is still in its early stages, and further investigations into the potential of miRNAs as biomarkers will be necessary in the future.

miR-146a, a member of the small, double-stranded, noncoding RNA family, plays a critical role as a negative regulator of inflammation and autoimmunity (83). In preclinical studies involving mice lacking miR-146a, the mice developed more severe irAEs than wild-type mice (84). This finding was verified by a clinical study in which patients with clear cell renal cell carcinoma who experienced grade 3 or 4 irAEs presented significantly reduced expression of exosomal miR-146a (85). Moreover, these two studies explored the impact of the *MIR146A* variant (rs2910164) on the severity of irAEs, consistently reporting that patients with the CC genotype were more likely to develop grade 3 or 4 irAEs. The role of exosomal miRNA-34a-5p in inducing cardiac senescence-related injury has also been revealed through experiments involving mouse models treated with PD-1 inhibitors (86). This finding supports the potential of miRNA-34a-5p as a biomarker for cardiac irAEs.

### The gut microbiome

The gut microbiome is a critical component of human physiology that influences both health and disorders such as inflammation, suggesting that it may impact irAE occurrence (87). Given that the gut microbiota might serve as a biomarker for irAEs, modifying it through interventions such as antibiotics, probiotics and fecal microbiota transplantation might offer a way to mitigate irAEs.

Nonetheless, the function of the gut microbiome as a biomarker for irAEs remains unclear because of inconsistent study results for numerous gut bacteria, which might not be easily reproducible and are difficult to interpret. For example, one study reported that patients with irAEs had reduced abundance of *Agathobacter*, though another study suggested that *Agathobacter* might be linked to more severe irAEs (88,89). As another limitation, collecting fecal samples from patients for analysis can be a tedious process.

### Body mass index

In recent years, several studies have provided evidence indicating that a high body mass index (BMI, kg/m^2^) is a predictive biomarker for irAEs (90). In comparison to patients with normal weight, those with BMI of ≥25 kg/m^2^ have a higher occurrence of irAEs of any grade. In addition, a retrospective study investigated the impact of weight and factors related to metabolic syndrome on irAEs, suggesting that individuals with overweight and few metabolic comorbidities are more likely to experience grade 2 or worse irAEs (91), though the mechanism remains unclear. Different metabolic statuses might exert opposite influences on PD-1 expression in T cells, resulting in complicated alterations in risk of irAEs.

### Future perspectives

With the rapid development and widespread use of ICIs, irAEs and their prediction have become a major concern. Despite considerable research efforts to explore potential biomarkers for irAEs, there is no established biomarker that can be used in routine clinical practice. This situation can be attributed to several factors. On one hand, routine laboratory tests, such as blood cell counts, ratios and serum proteins, are simple, feasible and cost-effective. However, studies investigating these markers as irAE predictors are largely retrospective in nature. On the other hand, evaluating complicated biological parameters such as cytokines/chemokines, genetic variations and gene expression is expensive and often not clinically available. Moreover, findings supporting their use as predictive biomarkers are sometimes inconsistent and lack specificity due to cancer-related inflammation and cancer development (92). This may contribute to the uncertainty of these biomarkers in predicting irAEs. Given these limitations, drawing definitive conclusions about the predictive value of these biomarkers for irAEs remains challenging. More large-scale prospective studies are required to confirm their ability to predict irAEs. Additional investigations are essential to demonstrate efficacy and subsequently facilitate integration into routine clinical use.

It is known that the development of irAEs is associated with better outcomes after ICI therapy across various types of cancers (93). It is quite plausible that biomarkers for irAEs might also predict the efficacy of ICI. For instance, baseline serum cytokine levels might predict the development of irAEs as well as the efficacy of ICI in NSCLC patients (56). Therefore, future research needs to go beyond merely predicting irAEs and, instead, explore ideal biomarkers that can simultaneously predict a lower risk of irAEs and higher treatment efficacy.

## Conclusion

Biomarkers that can predict the occurrence and severity of irAEs play a pivotal role in enhancing our ability to predict and manage these adverse events. Early identification and intervention are crucial for minimizing the impact of irAEs on patients undergoing ICI therapies. Many potential biomarkers have been proposed, but their sensitivity and specificity need to undergo validation through further studies before they can be confidently applied in various clinical settings.

## Funding

This work was financially supported by JST SPRING (Grant Number JPMJSP2125). Yao Liang would like to take this opportunity to thank the ‘Interdisciplinary Frontier Next-Generation Researcher Program of the Tokai Higher Education and Research System.’

## Conflict of interest statement

Dr Yuichi Ando reports grants and personal fees from Chugai Pharmaceutical Co, Ltd, grants and personal fees from Kyowa Kirin Co, Ltd, grants and personal fees from Nippon Kayaku Co, Ltd, grants and personal fees from Yakult Honsha Co, Ltd, personal fees from Eli Lilly Japan K.K., personal fees from Ono Pharmaceutical Co, Ltd, grants and personal fees from Taiho Pharmaceutical Co, Ltd, grants and personal fees from Novartis Pharma K.K., personal fees from Bayer Holding Ltd, personal fees from Sawai Pharmaceutical Co., Ltd, grants and personal fees from Daiichi Sankyo Company, Ltd, grants from Eisai Co, Ltd, personal fees from MSD K.K., personal fees from Astellas Pharma Inc, personal fees from Otsuka Holdings Co, Ltd, personal fees from Sanwa Kagaku Kenkyusho Co, Ltd, personal fees from Hisamitsu Pharmaceutical Co, Inc, personal fees from SymBio Pharceuticals, personal fees from Aptitude Health, grants from BeiGene, Ltd, personal fees from Alfresa Pharma Corporation, personal fees from Sumitomo Pharma Co. Sumitomo Pharma Co., Ltd, outside the submitted work.
